# Association of Systemic Immune-Inflammation Index With Short-Term Mortality of Congestive Heart Failure: A Retrospective Cohort Study

**DOI:** 10.3389/fcvm.2021.753133

**Published:** 2021-11-12

**Authors:** Yiyang Tang, Xiaofang Zeng, Yilu Feng, Qin Chen, Zhenghui Liu, Hui Luo, Lihuang Zha, Zaixin Yu

**Affiliations:** ^1^Department of Cardiology, Xiangya Hospital, Central South University, Changsha, China; ^2^Department of Neurology, Xiangya Hospital, Central South University, Changsha, China; ^3^Department of Cardiovascular Medicine, the First Hospital of Changsha, Changsha, China; ^4^National Clinical Research Center for Geriatric Disorders (Xiang Ya), Changsha, China

**Keywords:** congestive heart failure, systemic immune-inflammation index, mortality, biomarker, MIMIC-III database

## Abstract

**Purpose:** The present study aimed to clarify the potential predictive significance of Systemic immune-inflammation index (SII) in assessing the poor prognosis of critically ill patients with congestive heart failure (CHF).

**Methods:** Detailed clinical data were extracted from the Multiparameter Intelligent Monitoring in Intensive Care III database after gaining access and building the local platform. The 30- and 90-day and hospital all-cause mortalities of the patient was the primary outcome, and the readmission rate and the occurrence of major cardiovascular adverse events (MACEs) were the secondary outcomes. the Cox proportional hazard model and Logistic regression analysis were selected to reveal the relationship between SII level and the research outcome. Further, the propensity score matching (PSM) analysis was performed to improve the reliability of results by reducing the imbalance across groups.

**Results:** There were a total of 4,606 subjects who passed the screening process and entered the subsequent analysis. Multivariate regression analysis showed that after adjusting for possible confounders, including age, heart rate, and albumin, etc., the high level of SII was independently associated with 30- and 90-day and hospital mortalities (tertile 3 vs. tertile 1: HR, 95% CIs: 1.23, 1.04-1.45; 1.21, 1.06-1.39; 1.26, 1.05-1.50) and the incidence of MACEs (tertile 3 vs. tertile 1: OR, 95% CI: 1.39, 1.12-1.73) in critically ill patients with CHF, but no significant correlation was found between SII and the readmission rate. Consistently, patients with high SII level still presented a significantly higher short-term mortality than patients with low SII in the PSM subset.

**Conclusion:** In critically ill patients with CHF, high level of SII could effectively predict high 30- and 90-day and hospital mortalities, as well as the high risk of occurrence of MACEs.

## Introduction

Congestive heart failure (CHF) is defined as a pathological condition in which the cardiac output is insufficient to maintain the perfusion and metabolic needs of various tissues and organs, mainly characterized by the congestion of pulmonary circulation, accompanied by the corresponding clinical manifestations such as shortness of breath and decreased activity tolerance (1). CHF is the severe or terminal stage of most primary cardiovascular diseases and has increasingly become a major public problem threatening human health. In the United States and Europe, more than 1 million heart failure patients are hospitalized each year, and as the population ages, this number is expected to increase by more than 50% in the next 15 years (2). In addition, although some progress has been made in the treatment in recent years, the prognosis of CHF patients is still poor, with 5-year mortality rate as high as 40-50% (3). Therefore, accurate assessment and stratification of prognosis are critical to the clinical management of CHF, and the development of certain prognostic-related biomarkers is also pressing, which can help clinicians to identify high-risk patients early and take more aggressive treatment measures (4).

Although the specific pathogenesis of heart failure remains unclear, abnormal immune activation and chronic inflammation play an important role. The damage, repair and remodeling of myocardium are the important link in the occurrence and development of heart failure, of which immune/inflammatory cells (neutrophils, lymphocytes, etc.) and the inflammatory factors (tumor necrosis factor, interleukin-6, etc.) released by them are involved (5). Inflammatory factors can induce cardiomyocyte hypertrophy, apoptosis and fibrosis, and ultimately lead to adverse cardiac remodeling and progressive left ventricular dysfunction (6). Inflammatory factors have been considered to be biomarkers for poor prognosis of heart failure, and anti-inflammatory therapy is also expected to become a new target for the treatment of heart failure (7).

Systemic immune-inflammation index (SII) is a composite inflammatory indicator that combines three significant immune cells, including neutrophil, lymphocyte, and platelet, and is considered to be an excellent indicator of local immune response and systemic inflammation (8). Neutrophils, platelets and the cytokines they produce are mainly related to non-specific immune responses, while lymphocytes are considered to be mainly related to specific immune pathways. Compared with the absolute count of single immune cells, SII has a better representation in reflecting the inflammation status of the body, with better stability. Until now, SII has been confirmed to be closely correlated with the poor prognosis of a variety of cardiovascular diseases, including coronary artery disease (9), aortic stenosis (10), infective endocarditis (11), etc., showing good application prospects, but no studies have shown that SII is correlated with the prognosis of patients with CHF. In view of this, the present study attempted to elucidate the independent association between SII and the occurrence of adverse events such as short-term mortality in critically ill patients with CHF, so as to provide reference for the prevention and treatment of heart failure.

## Materials and Methods

### Data Sources

All data used in the research and analysis of this study are derived from MIMIC-III (Medical Information Mart for Intensive Care III) database, a free and open-access large-scale critical care medicine database developed and run by the Massachusetts Institute of Technology for researchers all over the world. Another advantage of this database, in addition to including the detailed clinical data such as demographic data, vital signs, laboratory tests, and various treatment information, is that it provides accurate death information of all subjects, including time of death in the hospital or within 90 days after discharge, which makes it possible for the clinicians to carry out the prognostic related research. Besides, since this database has been approved by the local Ethics Committee as a whole and the individual identifying information of the research subjects has been deleted, this study no longer needs additional ethical approval.

### Population Selection and Exclusion

The subjects of this study are all from the MIMIC- III database (12), and patients who meet all the following requirements are included in the subsequent analysis: (1) Patients diagnosed with CHF based on the ninth revision of the International Classification of Diseases (ICD-9) code (code 428.0); (2) Adult patients 18 years of age and older; (3) First admission to intensive care units (ICU). Patients who met one of the following criteria were excluded from the study: (1) The length of ICU stay was shorter than 24 h; (2) Absence of SII results during hospitalization; (3) Survival time was <0 (the time of death of some organ donors may be earlier than the admission).

### Study Outcomes

The short-term mortality, including the 30- and 90-day and hospital all-cause mortalities of critically ill patients with CHF, were selected as the primary study outcome, while the secondary study outcome events were defined as the patients' readmission rate and the occurrence of major adverse cardiac events (MACEs), which is a composite outcome event, including all-cause death, readmission for acute heart failure, the use of mechanical circulatory support and the implementation of heart transplantation (13).

### Data Extraction and Integration

Through the PostgreSQL software (version 9.6, https://www.postgresql.org/), we extracted detailed clinical data of the research subjects from the MIMIC-III database, including demographic data (age, gender, race, etc.), comorbidities (hypertension, diabetes mellitus, atrial fibrillation, etc.), vital signs (heart rate, blood pressure, respiratory rate, etc.), severity of illness scores [the sequential organ failure assessment (SOFA) and the simplified acute physiology score II (SAPSII) scores], laboratory tests (blood routine, electrolytes, etc.) and intervention measures (dialysis, mechanical ventilation, etc.). And SII was calculated as follows: SII = platelet × neutrophil/lymphocyte (14).

After separately extracting the required tables from the MIMIC-III database, the Stata software (version 16, https://www.stata.com/) was used to process and merge these original table and generate a complete table that can be used for subsequent analysis. Use the “winsorize” function to identify and process outliers, and fill in missing values through multiple compensation methods.

### Statistical Analyses

The continuous variables were presented in the form of mean ± standard deviation (SD) or median (interquartile range). If continuous variables satisfy both normal distribution and homogeneity of variance, *t*-test was used for analysis, while the Mann-Whitney U test was conducted if the normal distribution or homogeneity of variance were not satisfied. Categorical variables are expressed in the form of the number of cases (percentage), with the chi-square test (or Fisher's exact method) for analysis.

We constructed a generalized additive model (GAM) to determine the non-linear relationship between SII and 30- and 90-day all-cause mortalities in critically ill patients with CHF. In addition, we also visually show the relationship between SII and patients' survival through the Kaplan-Meier (K-M) curve, and use the Log-rank test for hypothesis testing.

In order to relieve the interference of possible confounding factors on the results, we completed univariate and multivariate regression analysis to further clarify the relationship between SII and outcome variables. In the crude model, no variables were adjusted. In model I, the age, gender, and race variables were adjusted, while model II further adjusted other 12 variables on the basis of these variables of model II, including heart rate, blood urea nitrogen (BUN), albumin, troponin T (cTnT), N-terminal probrain natriuretic peptide (NT-proBNP), urine output of first day, cardiac index, the type of first ICU admission, the use of mechanical ventilation and vasopressor, pneumonia, and liver diseases. The selection of confounding factors follows the following principles (15): (1) A certain factor has an influence of more than 10% on the research variable; (2) Some factors may have a significant impact on the outcome variable based on past experience. Besides, we used the variance inflation factor (VIF) to test the multicollinearity between variables with 5 as the threshold, and the variables with a high degree of collinearity, serum chloride, were deleted to avoid over-fitting of the model. The Cox regression analysis was used to determine the relationship between SII and short-term mortalities in critically ill patients with CHF, while Logistic regression analysis was used to analyze the association between SII and readmission rates or MACEs.

To further enhance the credibility of our analysis, we performed the subgroup analysis and propensity score matching (PSM) analysis. First, By the subgroup analysis, determine whether the correlation between the SII and the high 30-day all-cause mortality in critically ill patients with CHF was consistent across various subgroups stratified mainly by comorbidities, and reflect the stability of SII as a prognostic marker. The optimal cut-off value of SII for the short-term mortality was determined by the X-tile (version 3.6.1, Yale University School of Medicine) software, and then the entire study population was divided into two groups, namely the high SII group and the low SII group. In this study, all variables with uneven distribution between the two groups of patients were included in the PSM model as covariates, and the corresponding propensity score was calculated by Logistic regression. Then the two groups of individuals with similar propensity scores were matched by 1:1 using nearest neighbor matching method with a caliper width of 0.05, and the histogram of the propensity score distribution was draw, which was carried out through the Stata software. Then, K-M curves were depicted and Cox regression analysis was conducted in the matching cohort after PSM, which further validated SII as an independent risk factor for poor prognosis in the critically ill patients with CHF.

The statistical analysis above was conducted by EmpowerStats software (version 2.20, http://www.empowerstats.com/cn/, X&Y solutions, Inc, Boston, MA) and R software (version 3.4.3). *p* < 0.05 (two-sided) was considered statistically significant. Besides, in the regression analysis, we corrected *p*-value with false discovery rate (FDR) to avoid the false positives with multiple comparisons and used the false discovery rate (FDR) of 0.05 as cutoff.

## Results

### Clinical Characteristics of Study Subjects

**Figure 1:**
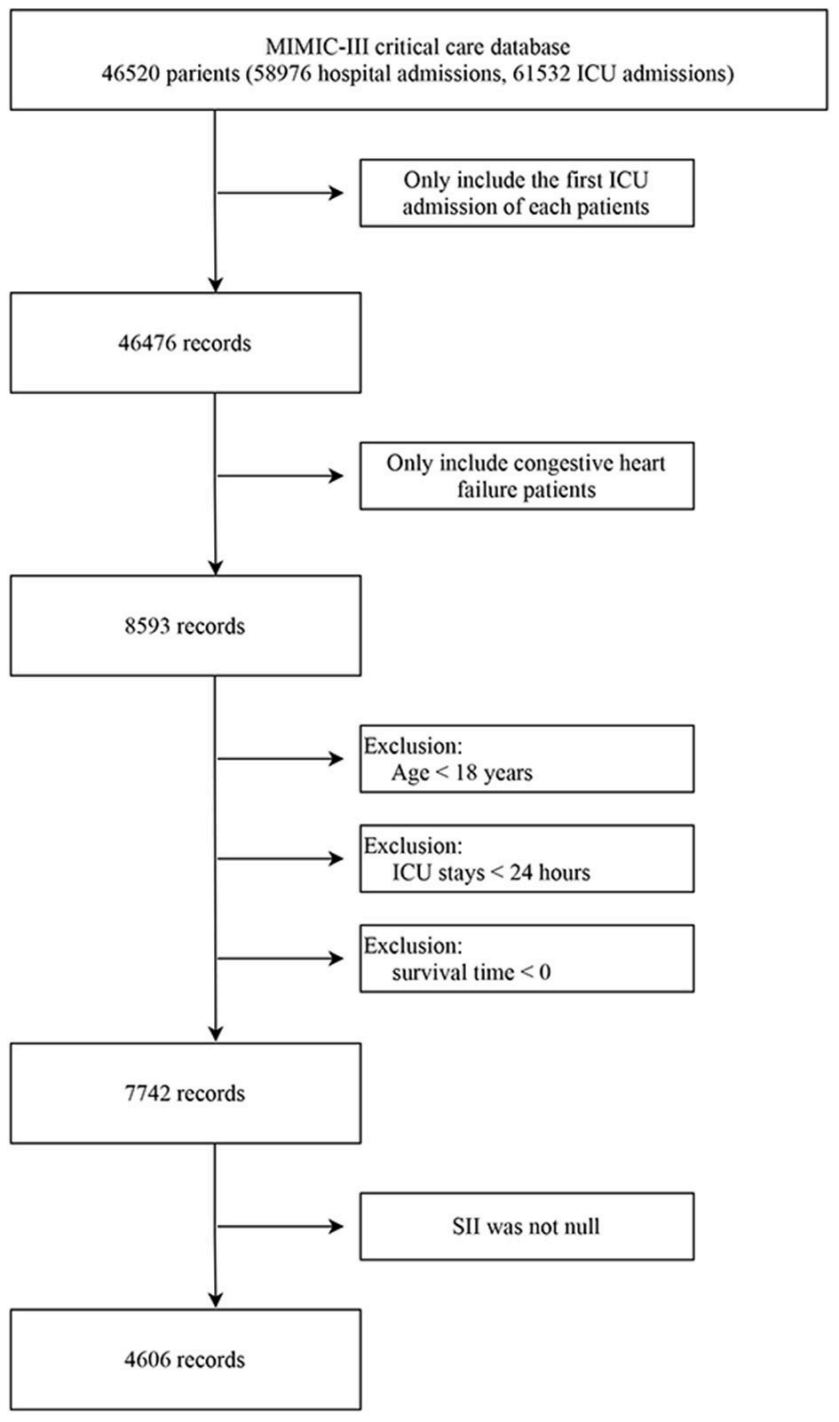
Workflow of exclusion and inclusion as utilized to select the final 4,606 patients. ICU, intensive care unit. SII, systemic immune-inflammation index.

The study subjects were screened according to the process mentioned above, and 4,606 subjects were finally included (Figure 1). The demographic data, vital signs, comorbidities, treatment, scores and laboratory examinations and other relevant information between survivors and non-survivors' cohorts were shown in Table 1 in detail. Besides, the clinical characteristics of critical ill patients with CHF across three group divided according to SII levels were presented in Supplementary Table 1. Overall, the median age of the study subjects included was 74.91 years old, mainly male patients, reaching 2,436, accounting for 52.89%. And the majority of the subjects were whites, accounting for 72.71%, blacks only 7.21%, and other races including Asians accounted for 20.08%. The duration of follow-up was 90 days, of which 3,226 survived and 1,380 died due to various reasons before the end of the study. SII (213.97 vs. 163.11, *p* < 0.001) was significantly higher in the non-survivor cohort. Compared with the survivor group, subjects in the non-survivor cohort were older (median age: 78.94 vs. 72.70 years old, *p* < 0.001), with more unstable vital signs, higher incidence of comorbidities such as valvular heart disease (VHD), atrial fibrillation, and renal failure, as well as higher SOFA and SAPSII scores.

**Table 1:** The baseline clinical characteristics of critically ill patients with CHF.

**Parameter**	**All (*n* = 4,606)**	**Survivors (*n* = 3,226)**	**Non-survivors (*n* = 1,380)**	***P*-value**
**Demographics**				
Age, years	74.91 (64.06-83.07)	72.70 (61.97-81.65)	78.94 (69.40-84.82)	<0.001
Male, *n* (%)	2436 (52.89%)	1690 (52.39%)	746 (54.06%)	0.298
Ethnicity, *n* (%)				<0.001
White	3349 (72.71%)	2361 (73.19%)	988 (71.59%)	
Black	332 (7.21%)	254 (7.87%)	78 (5.65%)	
Others	925 (20.08%)	611 (18.94%)	314 (22.75%)	
**Vital signs**				
HR, beats/minute	85.02 (74.53-96.54)	84.33 (74.33-95.77)	86.67 (75.24-98.65)	<0.001
RR, times/minute	19.42 (16.87-22.38)	19.17 (16.76-21.91)	20.03 (17.24-23.45)	<0.001
SBP, mmHg	113.64 (104.46-125.96)	114.89 (105.50-126.98)	111.04 (101.60-123.48)	<0.001
DBP, mmHg	56.93 (50.87-63.71)	57.85 (51.64-64.34)	55.10 (49.46-61.64)	<0.001
Temperature, °C	36.76 (36.37-37.21)	36.80 (36.42-37.22)	36.65 (36.26-37.13)	<0.001
SpO2, %	97.23 (95.74-98.46)	97.25 (95.81-98.49)	97.19 (95.52-98.40)	0.018
Weight, kg	77.75 (64.70-93.60)	79.80 (65.60-96.00)	73.80 (62.00-88.00)	<0.001
**Therapies, n (%)**				
ACEI	1266 (27.49%)	1012 (31.37%)	254 (18.41%)	<0.001
ARB	183 (3.97%)	151 (4.68%)	32 (2.32%)	<0.001
β-blocker	2827 (61.38%)	2074 (64.29%)	753 (54.57%)	<0.001
Digoxin	475 (10.31%)	303 (9.39%)	172 (12.46%)	0.002
Furosemide	3190 (69.26%)	2275 (70.52%)	915 (66.30%)	0.004
Statins	1451 (31.50%)	1121 (34.75%)	330 (23.91%)	<0.001
Dialysis	478 (10.38%)	278 (8.62%)	200 (14.49%)	<0.001
Vasopressor	1234 (26.79%)	727 (22.54%)	507 (36.74%)	<0.001
Ventilation	1879 (40.79%)	1133 (35.12%)	746 (54.06%)	<0.001
Assisted circulation	282 (6.12%)	186 (5.77%)	96 (6.96%)	0.123
**Laboratory events**				
Hemoglobin, g/dl	10.90 (9.70-12.30)	11.10 (9.80-12.50)	10.70 (9.40-11.90)	<0.001
Creatinine, mg/dl	1.20 (0.90-1.80)	1.20 (0.90-1.70)	1.30 (0.90-2.10)	<0.001
BUN, mg/dl	27.00 (18.00-43.00)	25.00 (17.00-40.00)	33.00 (21.00-51.00)	<0.001
Glucose, mg/dl	135.33 (115.00-166.12)	134.23 (115.47-164.03)	137.71 (114.36-170.52)	0.111
Sodium, mmol/L	139.00 (136.00-141.00)	139.00 (136.00-141.00)	138.00 (135.00-141.00)	0.023
Potassium, mmol/L	4.20 (3.80-4.60)	4.10 (3.70-4.60)	4.20 (3.80-4.70)	0.002
Chloride, mmol/L	103.00 (99.00-107.00)	103.00 (99.25-107.00)	103.00 (99.00-107.00)	0.035
Bicarbonate, mmol/L	24.00 (21.00-28.00)	25.00 (22.00-28.00)	24.00 (20.00-27.00)	<0.001
PT, second	14.30 (13.20-16.80)	14.20 (13.10-16.50)	14.80 (13.40-17.90)	<0.001
APTT, second	31.80 (27.30-40.40)	31.30 (27.10-40.20)	32.70 (27.87-41.10)	0.001
Lactate, mmol/L	1.82 (1.30-2.90)	1.80 (1.20-2.83)	2.00 (1.30-3.00)	<0.001
Albumin, g/dl	3.00 (2.56-3.40)	3.00 (2.60-3.42)	2.89 (2.45-3.24)	<0.001
Bilirubin, mg/dl	1.40 (0.60-3.05)	1.40 (0.60-3.00)	1.41 (0.60-3.10)	0.339
ALT, IU/L	60.00 (22.00-298.33)	69.00 (23.92-327.15)	45.00 (19.00-207.75)	<0.001
NT-proBNP, ng/ml	10.98 (4.67-18.83)	10.70 (4.26-18.34)	11.91 (5.67-19.96)	<0.001
cTnT, ng/ml	1.34 (0.10-2.87)	1.46 (0.11-2.94)	1.12 (0.09-2.58)	<0.001
CI, L/min/m^2^	2.76 (2.13-3.47)	2.75 (2.13-3.48)	2.78 (2.14-3.45)	0.626
Urine output, L	1.54 (0.89-2.44)	1.73 (1.02-2.65)	1.14 (0.67-1.87)	<0.001
SII × 10	174.76 (92.24-344.55)	163.11 (88.62-309.02)	213.97 (105.80-430.34)	<0.001
**Hospitalization type**				<0.001
Emergency, *n* (%)	4062 (88.19%)	2804 (86.92%)	1258 (91.16%)	
Elective, *n* (%)	367 (7.97%)	300 (9.30%)	67 (4.86%)	
First ICU admission				<0.001
CCU, *n* (%)	1236 (26.83%)	898 (27.84%)	338 (24.49%)	
MICU, *n* (%)	2005 (43.53%)	1308 (40.55%)	697 (50.51%)	
**Comorbidities**, ***n*** **(%)**				
Hypertension	937 (20.34%)	655 (20.30%)	282 (20.43%)	0.919
Hyperlipemia	1260 (27.36%)	975 (30.22%)	285 (20.65%)	<0.001
Diabetes mellitus	1657 (35.97%)	1218 (37.76%)	439 (31.81%)	<0.001
Atrial fibrillation	2077 (45.09%)	1361 (42.19%)	716 (51.88%)	<0.001
AMI	346 (7.51%)	241 (7.47%)	105 (7.61%)	0.871
VHD	463 (10.05%)	289 (8.96%)	174 (12.61%)	<0.001
Peripheral vascular	577 (12.53%)	397 (12.31%)	180 (13.04%)	0.489
Pulmonary circulation	292 (6.34%)	183 (5.67%)	109 (7.90%)	0.005
Pneumonia	1350 (29.31%)	858 (26.60%)	492 (35.65%)	<0.001
COPD	245 (5.32%)	156 (4.84%)	89 (6.45%)	0.025
Liver diseases	232 (5.04%)	138 (4.28%)	94 (6.81%)	<0.001
Renal failure	1120 (24.32%)	757 (23.47%)	363 (26.30%)	0.040
Stroke	250 (5.43%)	148 (4.59%)	102 (7.39%)	<0.001
Hypothyroidism	555 (12.05%)	392 (12.15%)	163 (11.81%)	0.746
Malignancy	253 (5.49%)	128 (3.97%)	125 (9.06%)	<0.001
Depression	359 (7.79%)	276 (8.56%)	83 (6.01%)	0.003
**Scores**				
SOFA	5.00 (3.00-7.00)	4.00 (3.00-6.00)	6.00 (4.00-8.00)	<0.001
SAPSII	40.00 (32.00-49.00)	38.00 (30.00-46.00)	47.00 (39.00-56.00)	<0.001
**Length of ICU stay, h**	94.00 (50.00-187.00)	88.00 (48.00-166.75)	122.00 (62.00-240.25)	<0.001

*HR, heart rate; RR, respiratory rate; SBP, systolic blood pressure; DBP, diastolic blood pressure; SpO2, percutaneous oxygen saturation; ACEI, angiotensin-converting enzyme inhibitors; ARB, angiotensin receptor blockers; BUN, blood urea nitrogen; PT, prothrombin time; APTT, activated partial thromboplastin time; ALT, alanine transaminase; cTnT, troponin T; NT-proBNP, N-terminal pro brain natriuretic peptide; CI, cardiac index; SII, Systemic immune-inflammation index; CHF, congestive heart failure; AMI, acute myocardial infarction; VHD, valvular heart disease; COPD, chronic obstructive pulmonary disease. CCU, cardiac care unit; MICU, medical intensive care unit*.

### SII Distinguishing a Poor Short-Term Prognosis

**Figure 2:**
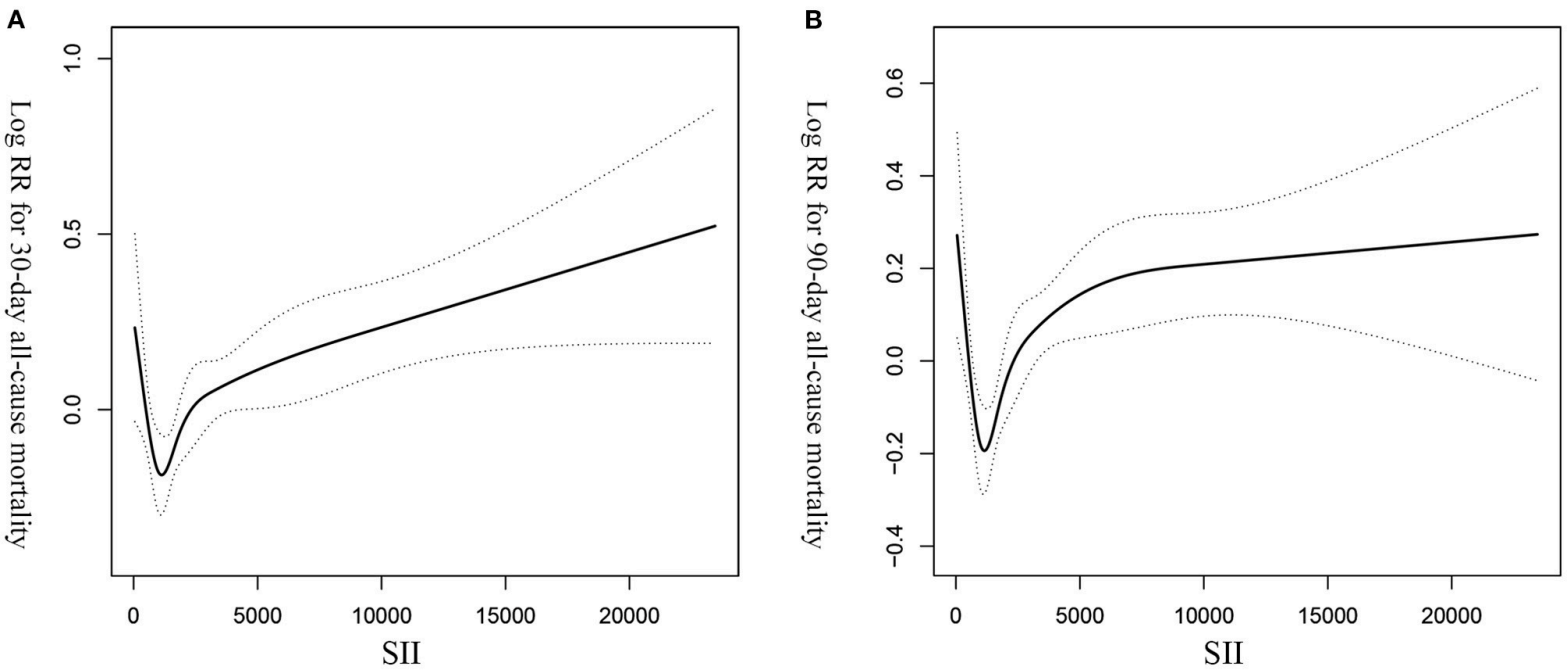
The non-linear curve fitting of the association between SII and 30- **(A)** and 90- **(B)** day all-cause mortalities in critically ill patients with CHF after adjusting for age, gender, race, and other potential variables. SII, systemic immune-inflammation index. CHF, congestive heart failure.

**Figure 3:**
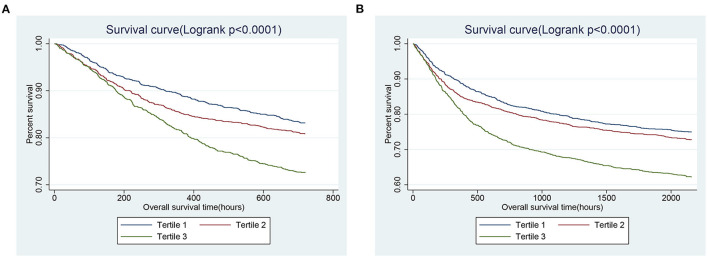
Kaplan-Meier survival curves showing the 30- **(A)** and 90- **(B)** day all-cause mortalities stratified by SII tertile in critically ill patients with CHF. SII, systemic immune-inflammation index. CHF, congestive heart failure.

GAM analysis was used to identify the non-linear correlation between SII and the short-term all-cause mortality of patients, which showed that there was a U-shaped curve relationship between SII and 30-day (Figure 2A) and 90-day (Figure 2B) all-cause mortalities in critically ill patients with CHF, indicating that higher or lower SII may be associated with increased mortality. The K-M survival curve (Figure 3) also showed that compared with the low SII group, patients with high SII level had a lower overall survival rate and shorter survival time, with a statistically significant *p*-value.

In order to verify the independent relationship between SII and the poor short-term mortality of CHF patients, we performed the univariate and multivariate Cox regression analysis (Table 2), with SII stratified by tertiles. In crude model, the third tertile of SII increased significantly the risk of 30- (HR, 95% CI: 1.73, 1.48-2.03) and 90-day (HR, 95% CI: 1.65, 1.45-1.88) and hospital (HR, 95% CI: 1.61, 1.36-1.91) all-cause mortalities compared with the first tertile. In multivariate model I, after adjusting for age, sex, and race, the third tertile of SII group also suffered from the higher risk of 30- (HR, 95% CI: 1.63, 1.40-1.91) and 90-day (HR, 95% CI: 1.57, 1.38-1.79) and hospital (HR, 95% CI: 1.45, 1.22-1.72) all-cause mortalities. In model II, in addition to adjusting for the variables in model I and other possible confounders such as pneumonia, the use of dialysis, and BUN level, high levels of SII were still strongly associated with poor 30- (HR, 95% CI: 1.23, 1.04-1.45) and 90-day (HR, 95% CI: 1.21, 1.06-1.39) and hospital (HR, 95% CI: 1.26, 1.05-1.50) all-cause mortalities.

**Table 2:** The univariate and multivariate Cox regression analysis exploring the association of SII tertile with short-term mortality of critically ill patients with CHF.

	**Crude**		**Model I**		**Model II**	
	**HR (95%CIs)**	**FDR**	**HR (95%CIs)**	**FDR**	**HR (95%CIs)**	**FDR**
**30-day all-cause mortality**						
**SII (tertile)**						
<1144.28	1 (ref)		1 (ref)		1 (ref)	
≥1144.28, <2730.11	1.17 (0.99, 1.38)	0.101	1.14 (0.97, 1.35)	0.134	1.05 (0.89, 1.25)	0.524
≥2730.11	1.73 (1.48, 2.03)	0.003	1.63 (1.40, 1.91)	0.003	1.23 (1.04, 1.45)	0.022
*P* for trend	<0.001		<0.001		<0.001	
**90-day all-cause mortality**						
**SII (tertile)**						
<1144.28	1 (ref)		1 (ref)		1 (ref)	
≥1144.28, <2730.11	1.12 (0.97, 1.28)	0.171	1.10 (0.96, 1.27)	0.202	1.02 (0.88, 1.17)	0.820
≥2730.11	1.65 (1.45, 1.88)	0.003	1.57 (1.38, 1.79)	0.003	1.21 (1.06, 1.39)	0.010
*P* for trend	<0.001		<0.001		<0.001	
**Hospital all-cause mortality**						
**SII (tertile)**						
<1144.28	1 (ref)		1 (ref)		1 (ref)	
≥1144.28, <2730.11	1.27 (1.06, 1.52)	0.016	1.21 (1.01, 1.45)	0.042	1.17 (0.97, 1.40)	0.096
≥2730.11	1.61 (1.36, 1.91)	0.003	1.45 (1.22, 1.72)	0.003	1.26 (1.05, 1.50)	0.017
*P* for trend	<0.001		<0.001		<0.001	

*Crude model was adjusted for none*.

*Model I was adjusted for age, gender, and race*.

*Model II was adjusted for age, gender, race, heart rate, blood urea nitrogen, albumin, troponin T, NT-proBNP, urine output of first day, cardiac index, the type of first ICU admission, the use of mechanical ventilation and vasopressor, pneumonia, and liver diseases*.

*SII, Systemic immune-inflammation index; CHF, congestive heart failure; HR, hazard ratio; CI, confidence interval. FDR, false discovery rate*.

### Association Between SII and Readmission and MACEs

For MACEs, a similar trend was observed. In model II (Table 3), patients with an SII ≥ 2730.11 were still at higher incidence of MACEs (OR, 95% CI: 1.39, 1.12-1.73). However, in the multivariate regression analysis, there was no obvious independent correlation between SII and readmission rate in the critically ill patients with CHF (tertile 3 vs. tertile 1: OR, 95% CI: 0.97, 0.81-1.15).

**Table 3:** The univariate and multivariate Logistic regression analysis exploring the association of SII tertile with readmission and MACEs of critically ill patients with CHF.

	**Crude**		**Model I**		**Model II**	
	**OR (95%CIs)**	**FDR**	**OR (95%CIs)**	**FDR**	**OR (95%CIs)**	**FDR**
**Readmission**						
**SII (tertile)**						
<1144.28	1 (ref)		1 (ref)		1 (ref)	
≥1144.28, <2730.11	0.91 (0.77, 1.07)	0.581	0.94 (0.80, 1.11)	0.581	0.94 (0.80, 1.11)	0.581
≥2730.11	0.88 (0.75, 1.04)	0.581	0.93 (0.78, 1.10)	0.581	0.97 (0.81, 1.15)	0.703
*P* for trend	<0.001		<0.001		<0.001	
**MACEs**						
**SII (tertile)**						
<1144.28	1 (ref)		1 (ref)		1 (ref)	
≥1144.28, <2730.11	1.17 (1.02, 1.35)	0.036	1.17 (1.02, 1.35)	0.036	0.92 (0.74, 1.13)	0.417
≥2730.11	2.01 (1.73, 2.32)	0.003	2.02 (1.74, 2.35)	0.003	1.39 (1.12, 1.73)	0.006
*P* for trend	<0.001		<0.001		<0.001	

*Crude model was adjusted for none*.

*Model I was adjusted for age, gender, and race*.

*Model II was adjusted for age, gender, race, heart rate, blood urea nitrogen, albumin, troponin T, NT-proBNP, urine output of first day, cardiac index, the type of first ICU admission, the use of mechanical ventilation and vasopressor, pneumonia, and liver diseases*.

*SII, Systemic immune-inflammation index; CHF, congestive heart failure; OR, odds ratio; CI, confidence interval. FDR, false discovery rate*.

### Subgroup Analyses

The subgroup analysis was conducted to reveal the correlation between SII and 30-day mortality across comorbidities and different parameters, and the results were shown in Table 4. First, the results of the study showed that in all subgroups, the increase in SII level was closely related to the increase in the 30-day all-cause mortality of critically ill patients with CHF. Besides, most of the stratification factors have not been found to have a significant impact on the relationship between the SII and 30-day all-cause mortality (interaction *p*-value > 0.05), except for hyperlipemia (*p* = 0.006) and acute myocardial infarction (AMI, *p* = 0.016).

**Table 4:** Subgroup analysis of the relationship between SII and 30-day all-cause mortality.

	***N***	**SII**			***P* for interaction**
		**<1144.28**	**≥1144.28, <2730.11**	**≥2730.11**	
Hypertension					0.178
No	3,669	1.0 (ref)	1.09 (0.90, 1.31)	1.70 (1.44, 2.02)	
Yes	937	1.0 (ref)	1.57 (1.07, 2.31)	1.88 (1.28, 2.76)	
Diabetes					0.384
No	2,949	1.0 (ref)	1.16 (0.95, 1.42)	1.62 (1.34, 1.95)	
Yes	1,657	1.0 (ref)	1.22 (0.90, 1.65)	2.01 (1.52, 2.67)	
Hyperlipemia					0.006
No	3,346	1.0 (ref)	1.13 (0.94, 1.36)	1.51 (1.27, 1.80)	
Yes	1,260	1.0 (ref)	1.40 (0.96, 2.05)	2.77 (1.94, 3.96)	
AMI					0.016
No	4,260	1.0 (ref)	1.09 (0.92, 1.30)	1.71 (1.46, 2.01)	
Yes	346	1.0 (ref)	2.63 (1.30, 5.30)	2.31 (1.14, 4.69)	
VHD					0.602
No	4143	1.0 (ref)	1.15 (0.96, 1.38)	1.76 (1.49, 2.07)	
Yes	463	1.0 (ref)	1.23 (0.76, 2.00)	1.50 (0.94, 2.41)	
PCD					0.931
No	4,314	1.0 (ref)	1.17 (0.98, 1.39)	1.72 (1.46, 2.02)	
Yes	292	1.0 (ref)	1.15 (0.61, 2.19)	1.86 (1.05, 3.32)	
Atrial fibrillation					0.437
No	2,529	1.0 (ref)	1.04 (0.82, 1.31)	1.59 (1.28, 1.98)	
Yes	2,077	1.0 (ref)	1.29 (1.02, 1.64)	1.84 (1.47, 2.31)	
Pneumonia					0.061
No	3,256	1.0 (ref)	1.18 (0.96, 1.44)	1.89 (1.56, 2.29)	
Yes	1,350	1.0 (ref)	1.09 (0.82, 1.45)	1.33 (1.03, 1.74)	
COPD					0.962
No	4,361	1.0 (ref)	1.16 (0.98, 1.38)	1.72 (1.47, 2.02)	
Yes	245	1.0 (ref)	1.32 (0.54, 3.20)	1.86 (0.83, 4.17)	
Liver diseases					0.065
No	4,374	1.0 (ref)	1.26 (1.06, 1.50)	1.85 (1.57, 2.18)	
Yes	232	1.0 (ref)	0.62 (0.34, 1.13)	1.24 (0.70, 2.21)	
Renal failure					0.396
No	3,486	1.0 (ref)	1.10 (0.91, 1.33)	1.72 (1.44, 2.05)	
Yes	1,120	1.0 (ref)	1.39 (1.00, 1.94)	1.79 (1.30, 2.48)	
Hypothyroidism					0.628
No	4,051	1.0 (ref)	1.13 (0.95, 1.35)	1.71 (1.45, 2.01)	
Yes	555	1.0 (ref)	1.46 (0.90, 2.38)	1.96 (1.23, 3.12)	
Stroke					0.618
No	4,356	1.0 (ref)	1.14 (0.96, 1.36)	1.69 (1.44, 1.99)	
Yes	250	1.0 (ref)	1.45 (0.79, 2.65)	2.24 (1.26, 3.97)	
Depression					0.216
No	4,247	1.0 (ref)	1.17 (0.98, 1.39)	1.79 (1.52, 2.10)	
Yes	359	1.0 (ref)	1.11 (0.55, 2.25)	1.02 (0.51, 2.03)	
Malignancy					0.879
No	4,353	1.0 (ref)	1.18 (0.99, 1.40)	1.72 (1.46, 2.02)	
Yes	253	1.0 (ref)	1.02 (0.57, 1.83)	1.57 (0.94, 2.63)	
SOFA					0.172
<5	2,149	1.0 (ref)	1.24 (0.91, 1.70)	2.21 (1.66, 2.96)	
≥5	2,457	1.0 (ref)	1.20 (0.99, 1.46)	1.58 (1.31, 1.90)	
SAPSII					0.255
<40	2,181	1.0 (ref)	1.22 (0.87, 1.71)	1.99 (1.43, 2.75)	
≥40	2,425	1.0 (ref)	1.13 (0.93, 1.37)	1.42 (1.19, 1.69)	

*SII, Systemic immune-inflammation index; AMI, acute myocardial infarction; VHD, valvular heart disease; PCD, peripheral vascular diseases; COPD, chronic obstructive pulmonary disease*.

### Prognostic Value of SII After PSM

**Figure 4:**
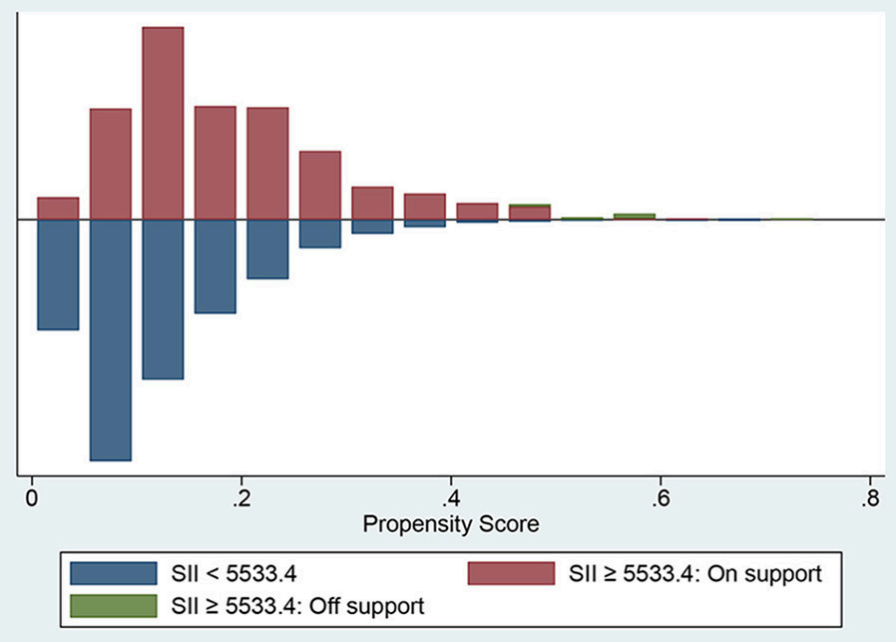
The histogram presenting the range of propensity scores and the corresponding number of matches in high (red) and low (blue) SII groups. SII, systemic immune-inflammation index.

**Figure 5:**
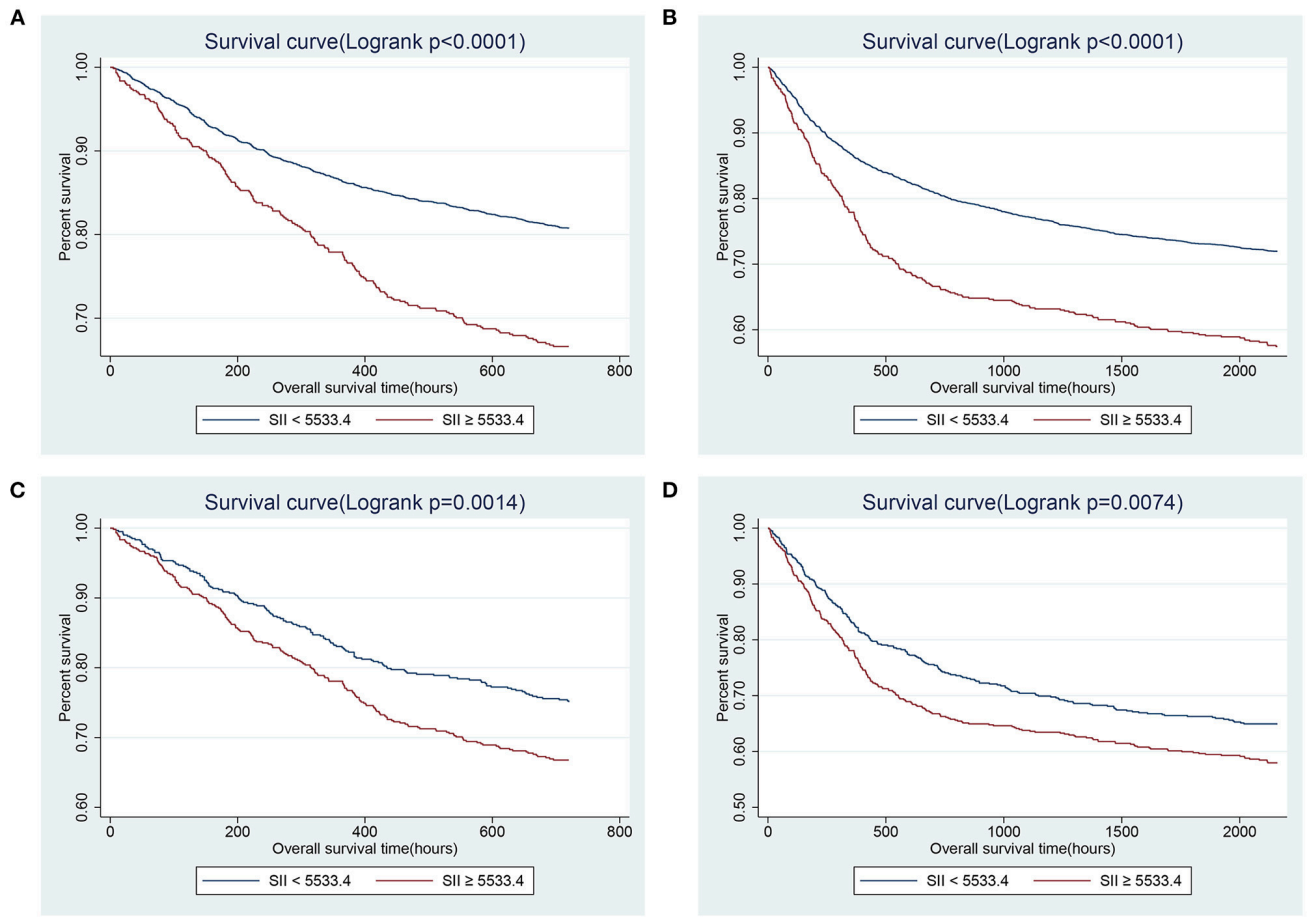
Kaplan-Meier survival curves showing the 30- **(A)** and 90- **(B)** day all-cause mortalities stratified by cut-off value of SII in critically ill patients with CHF before PSM. **(C,D)** presented the correlation between SII and 30- and 90-day mortalities after PSM, separately. SII, systemic immune-inflammation index. CHF, congestive heart failure. PSM, propensity score matching.

**Figure 6:**
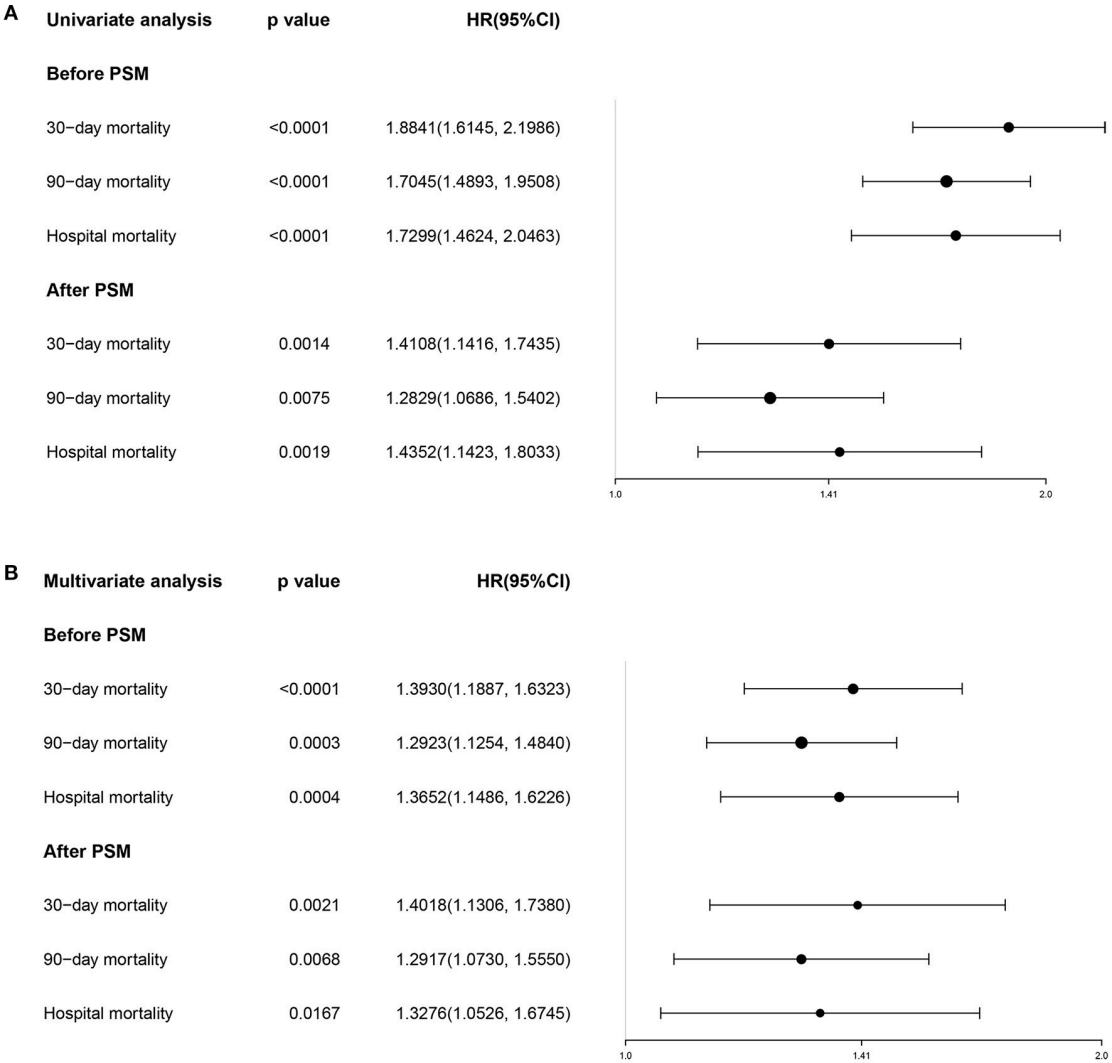
The forest plot showing the univariate **(A)** and multivariate **(B)** Cox regression for effects of SII on short-term mortality in the critically ill patients with CHF before and after PSM. SII, systemic immune-inflammation index. CHF, congestive heart failure. PSM, propensity score matching.

Determine the optimal cut-off value of SII for 90-day all-cause mortality of critically ill patients with CHF through X-tile software, which is 5533.4. Subjects were divided into two groups according to the cut-off value, and their clinical characteristics were summarized in Table 5, most of which were unevenly distributed across the two groups. In order to effectively balance these confounding factors and improve the credibility of our results, we conducted a PSM analysis with 1:1 matching. After PSM, 602 pairs of research objects were generated and the difference of almost all variables were balanced between the two groups, with a good matching performance (Figure 4). At the same time, after PSM, compared with the low SII level group, subjects in the high SII level group still underwent higher 30- (24.9 vs. 33.2%, *p* = 0.002) and 90-day (35.0 vs. 42.0%, *p* = 0.013) and hospital (22.4 vs. 27.7%, *p* = 0.033) all-cause mortalities, with shorter survival time (Table 5; Figure 5). Besides, after adjusting for these covariable in the model II, the increased SII was also associated with increased risk of short-term all-cause mortality in critically ill patients with CHF after PSM and the results were shown in Figure 6 in the form of a forest map.

**Table 5:** The clinical characteristics in critically ill patients with CHF before and after PSM.

**Parameter**	**Before PSM**			**After PSM**		
	**<5533.4**	**≥5533.4**	***P* value**	**<5533.4**	**≥5533.4**	***P*-value**
*N*	3,995	611		602	602	602
**Demographics**						
Age, years	74.4 (63.4-82.9)	76.9 (68.2-83.7)	<0.001	77.7 (66.1-84.2)	76.9 (68.1-83.7)	0.984
Male, n (%)	2154 (53.9%)	282 (46.2%)	<0.001	279 (46.3%)	279 (46.3%)	1.000
Ethnicity, *n* (%)		0.001	0.001			0.526
White	2879 (72.1%)	470 (76.9%)		463 (76.9%)	461 (76.6%)	
Black	309 (7.7%)	23 (3.8%)		30 (5.0%)	23 (3.8%)	
Others	807 (20.2%)	118 (19.3%)		109 (18.1%)	118 (19.6%)	
**Vital signs**						
HR, beats/minute	84.5 (74.1-96.0)	88.2 (77.0-99.6)	<0.001	87.5 (77.3-98.8)	87.9 (76.8-99.2)	0.840
RR, times/minute	19.3 (16.8-22.3)	20.3 (17.6-23.2)	<0.001	20.2 (17.3-23.5)	20.2 (17.6-23.1)	0.876
SBP, mmHg	113.8 (104.6-126.0)	113.8 (104.6-126.0)	0.145	113.9 (103.4-125.6)	112.1 (103.5-125.7)	0.964
DBP, mmHg	57.1 (51.1-63.9)	55.7 (50.2-62.5)	0.009	55.3 (49.1-61.9)	55.6 (50.2-62.5)	0.219
Temperature, °C	36.8 (36.4-37.2)	36.7 (36.3-37.1)	0.010	36.8 (36.4-37.2)	36.7 (36.3-37.1)	0.254
SpO2, %	97.3 (95.8-98.5)	97.0 (95.5-98.4)	0.157	97.3 (95.6-98.5)	97.0 (95.5-98.4)	0.474
Weight, kg	78.4 (65.0-94.3)	74.4 (61.0-90.0)	<0.001	73.0 (61.0-88.5)	74.4 (61.0-90.0)	0.560
**Therapies**, ***n*** **(%)**						
ACEI	1117 (28.0%)	149 (24.4%)	0.065	153 (25.4%)	147 (24.4%)	0.689
ARB	158 (4.0%)	25 (4.1%)	0.872	26 (4.3%)	25 (4.2%)	0.886
β-blocker	2454 (61.4%)	373 (61.0%)	0.858	373 (62.0%)	367 (61.0%)	0.722
Digoxin	411 (10.3%)	64 (10.5%)	0.888	85 (14.1%)	63 (10.5%)	0.053
Furosemide	2766 (69.2%)	424 (69.4%)	0.937	412 (68.4%)	416 (69.1%)	0.804
Statins	1274 (31.9%)	177 (29.0%)	0.148	158 (26.2%)	174 (28.9%)	0.302
Dialysis	426 (10.7%)	52 (8.5%)	0.104	86 (14.3%)	51 (8.5%)	0.001
Vasopressor	1042 (26.1%)	192 (31.4%)	0.008	171 (28.4%)	187 (31.1%)	0.313
Ventilation	1561 (39.1%)	318 (52.0%)	<0.001	306 (50.8%)	310 (51.5%)	0.818
Assisted circulation	259 (6.5%)	23 (3.8%)	0.009	21 (3.5%)	23 (3.8%)	0.759
**Scores**						
SOFA	5.0 (3.0-7.0)	5.0 (3.0-7.0)	0.556	5.0 (3.0-7.0)	5.0 (3.0-7.0)	0.405
SAPSII	40.0 (32.0-49.0)	45.0 (36.0-53.5)	<0.001	44.0 (35.0-53.0)	45.0 (36.0-53.0)	0.440
**Laboratory events**						
Hemoglobin, g/dl	11.0 (9.7-12.4)	10.7 (9.5-12.1)	0.008	10.7 (9.5-11.9)	10.7 (9.5-12.1)	0.290
Creatinine, mg/dl	1.2 (0.9-1.8)	1.2 (0.9-2.1)	0.250	1.3 (0.9-2.0)	1.2 (0.9-2.1)	0.160
BUN, mg/dl	26.0 (18.0-42.0)	30.0 (19.0-49.0)	<0.001	31.0 (20.0-49.0)	30.0 (19.0-48.0)	0.613
Glucose, mg/dl	134.0 (114.4-163.6)	145.5 (119.7-176.8)	<0.001	141.2 (117.1-180.3)	145.5 (119.6-176.2)	0.610
Sodium, mmol/L	139.0 (136.0-141.0)	138.0 (135.0-141.0)	<0.001	138.0 (135.0-141.0)	138.0 (135.0-141.0)	0.487
Potassium, mmol/L	4.2 (3.8-4.6)	4.2 (3.7-4.7)	0.575	4.2 (3.8-4.7)	4.2 (3.7-4.7)	0.634
Chloride, mmol/L	103.0 (100.0-107.0)	102.0 (98.0-107.0)	<0.001	103.0 (99.0-106.0)	102.0 (98.0-107.0)	0.377
Bicarbonate, mmol/L	25.0 (21.0-28.0)	24.0 (20.0-27.0)	<0.001	24.0 (20.0-27.0)	24.0 (20.0-27.0)	0.878
PT, second	14.3 (13.2-16.8)	14.4 (13.2-17.1)	0.245	14.4 (13.2-17.3)	14.4 (13.2-17.2)	0.846
APTT, second	31.8 (27.3-41.1)	31.6 (27.3-38.6)	0.329	31.3 (27.3-40.2)	31.7 (27.3-38.7)	0.920
Lactate, mmol/L	1.9 (1.3-2.9)	1.8 (1.3-2.8)	0.654	1.8 (1.3-3.0)	1.8 (1.3-2.8)	0.654
Albumin, g/dl	3.0 (2.6-3.4)	2.9 (2.5-3.3)	<0.001	2.8 (2.4-3.3)	2.9 (2.5-3.3)	0.255
Bilirubin, mg/dl	1.5 (0.6-3.1)	0.9 (0.4-2.5)	<0.001	1.1 (0.5-2.4)	0.9 (0.4-2.6)	0.282
ALT, IU/L	62.0 (22.0-309.0)	50.0 (21.0-242.7)	0.034	51.0 (21.0-225.8)	50.0 (21.0-248.5)	0.637
NT-proBNP, ng/ml	10.9 (4.6-18.6)	12.1 (5.1-19.8)	0.019			
cTnT, ng/mL	1.4 (0.1-2.9)	1.1 (0.1-2.5)	0.016	1.1 (0.1-2.8)	1.1 (0.1-2.5)	0.768
CI, L/min/m^2^	2.8 (2.1-3.5)	2.8 (2.2-3.5)	0.309	2.8 (2.1-3.4)	2.8 (2.2-3.5)	0.938
Urine output, L	1.6 (0.9-2.5)	1.4 (0.8-2.1)	<0.001	1.4 (0.7-2.2)	1.4 (0.8-2.1)	0.522
**Hospitalization type**			0.002			0.238
Emergency, *n* (%)	3498 (87.6%)	564 (92.3%)		538 (89.4%)	555 (92.2%)	
Elective, *n* (%)	338 (8.5%)	29 (4.7%)		39 (6.5%)	29 (4.8%)	
**First ICU admission**			<0.001			0.004
CCU, *n* (%)	1098 (27.5%)	138 (22.6%)		143 (23.8%)	138 (22.9%)	
Length of ICU stay, h	93.0 (50.0-184.0)	109.0 (54.0-204.0)	0.003	118.5 (59.0-228.8)	109.0 (53.0-201.8)	0.128
**Comorbidities**, ***n*** **(%)**						
Atrial fibrillation	1783 (44.6%)	294 (48.1%)	0.107	281 (46.7%)	290 (48.2%)	0.603
AMI	301 (7.5%)	45 (7.4%)	0.882	39 (6.5%)	44 (7.3%)	0.570
VHD	402 (10.1%)	61 (10.0%)	0.952	88 (14.6%)	61 (10.1%)	0.018
Peripheral vascular	511 (12.8%)	66 (10.8%)	0.167	88 (14.6%)	64 (10.6%)	0.037
Pulmonary circulation	239 (6.0%)	53 (8.7%)	0.011	45 (7.5%)	52 (8.6%)	0.459
Pneumonia	1108 (27.7%)	242 (39.6%)	<0.001	235 (39.0%)	237 (39.4%)	0.906
COPD	188 (4.7%)	57 (9.3%)	<0.001	48 (8.0%)	53 (8.8%)	0.603
Liver diseases	214 (5.4%)	18 (2.9%)	0.011	20 (3.3%)	18 (3.0%)	0.742
Renal failure	982 (24.6%)	138 (22.6%)	0.284	162 (26.9%)	136 (22.6%)	0.083
Stroke	227 (5.7%)	23 (3.8%)	0.051	38 (6.3%)	23 (3.8%)	0.052
Hypothyroidism	461 (11.5%)	94 (15.4%)	0.007	97 (16.1%)	92 (15.3%)	0.692
Malignancy	209 (5.2%)	44 (7.2%)	0.047	31 (5.1%)	43 (7.1%)	0.150
Depression	303 (7.6%)	56 (9.2%)	0.175	50 (8.3%)	56 (9.3%)	0.542
Hypertension	830 (20.8%)	107 (17.5%)	0.062	126 (20.9%)	106 (17.6%)	0.144
Hyperlipemia	1120 (28.0%)	140 (22.9%)	0.008	133 (22.1%)	138 (22.9%)	0.730
Diabetes mellitus	1449 (36.3%)	208 (34.0%)	0.285	205 (34.1%)	205 (34.1%)	1.000
30-day mortality	769 (19.2%)	204 (33.4%)	<0.001	150 (24.9%)	200 (33.2%)	0.002
90-day mortality	1120 (28.0%)	260 (42.6%)	<0.001	211 (35.0%)	253 (42.0%)	0.013
Hospital-day mortality	659 (16.5%)	172 (28.2%)	<0.001	135 (22.4%)	167 (27.7%)	0.033

*PSM, propensity score matching; HR, heart rate; RR, respiratory rate; SBP, systolic blood pressure; DBP, diastolic blood pressure; SpO2, percutaneous oxygen saturation; ACEI, angiotensin-converting enzyme inhibitors; ARB, angiotensin receptor blockers; BUN, blood urea nitrogen; PT, prothrombin time; APTT, activated partial thromboplastin time; ALT, alanine transaminase; cTnT, troponin T; NT-proBNP, N-terminal pro brain natriuretic peptide; CI, cardiac index; SII, Systemic immune-inflammation index; CHF, congestive heart failure; AMI, acute myocardial infarction; VHD, valvular heart disease; COPD, chronic obstructive pulmonary disease. CCU, cardiac care unit*.

## Discussion

CHF is widespread all over the world, with gradually increasing incidence with age, and has become one of the diseases with the highest rates of hospitalizations and deaths in the world, bringing about enormous medical expenditure and social burden, especially in developing countries (16). In addition to exploring promising treatments, the development of novel prognostic markers for risk stratification of patients is also of great value for improving the prognosis of patients. As we all know, as a commonly used indicator of heart failure, NT-proBNP can make a good performance in prognostic evaluation. However, the clinical applications of NT-proBNP for risk assessment has some drawbacks and problems. First, compared to the real value, the detection of NT-proBNP tend to be underestimated because of the short half-life. Moreover, the test of NT-proBNP may be affected by various factors such as age, gender, and kits (17). Therefore, the search for more possible biomarkers has gradually attracted the attention of researchers and clinicians.

In the present study, we extracted the clinical data of 4,606 critically ill patients who diagnosed with CHF between 2001 and 2012 in MIMIC-III database, and analyzed the relationship between SII and short-term prognosis by univariate and multivariate regression analyses for the first time. And the results have verified that SII could be an independent biomarker for poor short-term prognosis of CHF. There was a non-linear relationship between SII and 30- and 90-day mortalities. And the higher SII level, the higher risk of 30- and 90-day and hospital mortalities, as well as the higher incidence of MACEs. The correlation remained significant after adjusting for possible confounders, stratifying according to comorbidities, and PSM matching, respectively.

Inflammation, a widely accepted hallmark of heart failure, has been proven to play an indispensable role in the pathogenesis of heart failure (18). The increase and activation of inflammation-related cytokines not only present a reflection of the activation of inflammation *in vivo*, but have also been identified as being associated with poor prognosis in heart failure. Tumor necrosis factor α (TNF-α) was the first cytokine found to be significantly elevated in the serum of patients with heart failure, which could directly induce myocardial apoptosis and necrosis, bringing about ventricular adverse remodeling (19). The elevated levels of TNF-α often indicated impaired cardiac systolic function and poor long-term survival (20). In addition, interleukin 6 (IL-6), another classic cytokine mainly derived from monocytes, could be significantly increased in patients with left ventricular systolic dysfunction without clinical symptoms, and may be a sensitive indicator for the early diagnosis of heart failure (21). IL-6 was also positively correlated with the severity of heart failure, the concentration of which was negatively correlated with left ventricular ejection fraction and overall survival (22). Although these inflammatory molecules had good performance in the prognostic evaluation of patients, the expensive detection cost limited their clinical application. As a novel biomarker, SII can systematically and comprehensively reflect the status of inflammation *in vivo*. Blood routine is a routine examination for almost all hospitalized patients. It is simple, fast, and inexpensive. Compared with NT-proBNP, cytokines and other prognostic indicators, SII can effectively screen high-risk patients, guide the formulation of individualized treatment plans and improve the prognosis of patients without additional cost, with a broad application prospect, especially in underdeveloped areas.

The mechanisms of the relationship between SII and adverse prognosis of CHF remains unclear, and possible explanations are as follows. Recently, it has been revealed that circulating immune cells and their subtypes had a vital indicative effect on the prognosis of cardiovascular diseases (23). SII was a prognostic indicator that integrates three circulating immune cells, including neutrophils, lymphocytes, and platelets, the increase of which indicates either a relative increase in platelets and neutrophils counts or a relative decrease in lymphocytes. Neutrophils are the main component of white blood cells, accounting for 60-70% of the total, and play an important role in the non-specific immune system. In the inflammatory response, neutrophils react rapidly, with strong chemotaxis and phagocytosis, and participate in the progression of various cardiovascular diseases, including heart failure (24). In addition, neutrophils could contribute to oxidative stress and endothelial dysfunction by releasing a large amount of myeloperoxidase, NADPH oxidase, and so on (25). According to a community-based study (26), absolute neutrophils can effectively predict the increased incidence of acute decompensated heart failure after AMI, and it was also an independent risk factor for death (27). Lymphocytes mainly consist of two subtype including T and B cells, which were often associated with the specific immunity. Several previous studies have reported that lymphocyte counts in patients with heart failure were lower than in the normal population and lymphopenia served as an independent predictor of poor survival of patients with chronic and advanced heart failure (28, 29). Lymphopenia was considered to be an important feature of systemic inflammation, which may be caused by the following mechanisms. First, the circulating lymphocytes were attracted into the cardiac tissue, leading to its redistribution. Then, lymphopenia was related to the activation of renin-angiotensin-aldosterone hormone and the adrenergic nervous system, which could exert pro-apoptotic effects on lymphocytes (28). Platelets were differentiated from the mononuclear-phagocyte system and were the key mediator linking the two pathological processes of inflammation and thrombosis (30). On the one hand, platelets were the main effector molecules for hemostasis after cardiovascular injury. On the other hand, the activation of platelets plays a major role in pathogenic thrombosis and participates in the pathogenesis of many groups of cardiovascular diseases, including coronary heart disease (31). Moreover, Platelets interacted with leukocytes and their subtypes (neutrophil, lymphocytes, etc.) and endothelial cells and activated them, inducing monocyte adhesion and transport, releasing inflammatory factors such as TNF-α and IL-1, which together promoted local inflammation and fibrosis in heart failure (31). The study by Kandis et al. (32) demonstrated that mean platelet volume (MPV), indicating the activation of platelets, increased significantly in decompensated heart failure patients. In addition, MPV at admission was independently associated with the hospital and 6-month mortalities.

Besides, the subgroup analysis was also conducted, and the results demonstrated that the association was stable and consistent between the high level of SII and the poor 30-day mortality across CHF patients with different comorbidities or severity scores. We also noted that, among the critically ill patients with CHF, patients complicated with AMI and hyperlipidemia had a higher risk of 30-day mortality, and this risk was higher for higher SII, which implied that SII may be more valuable for the prognostic evaluation of CHF patients with AMI or hyperlipidemia. Similarly, the previous study has also shown that in patients with coronary heart disease, the high level of SII indicated a high risk of cardiac death in the future, as well as the high incidence of non-fatal stroke and heart failure (9). Although there was no research on the correlation between SII and hyperlipidemia, the interconnection of hyperlipidemia and inflammation involved in the pathogenesis of cardiovascular disease has been recognized (33).

As a retrospective cohort study, the existence of confounding factors cannot be ignored, and it was difficult to intuitively and accurately judge the correlation between SII level and the prognosis of CHF patients. Another highlight of this study was the use of PSM analysis to reduce the imbalance of confounding factors across different groups. After PSM, the most baseline characteristics were well-balanced between these two groups, except for the use of dialysis, the type of first ICU admission, and the peripheral vascular diseases. More importantly, the conclusion that high levels of SII were independently related to high short-term mortality remained stand before and after PSM, which improved the reliability of SII as a prognostic marker of CHF.

But at the same time, there were certain limitations in this study. First, the present study was conducted at a single center and did not validate the prognostic value of SII in a validation cohort. Second, the clinical data were collected retrospectively from databases and it was difficult to ensure that variables were evenly distributed across groups, although multiple regression models were conducted to adjust for confounders and PSM analysis was used to minimize inter-group differences in baseline characteristics. Third, some significant variables may be omissive due to the lack of data. Last, this study was unable to determine the underlying mechanism of the association between high SII and poor prognosis of CHF patients, and further experiments are necessary.

## Conclusion

In summary, the high level of SII is closely related to the poor short-term prognosis in critically ill patients with CHF, including 30- and 90-day and hospital all-cause mortalities, as well as the occurrence of MACEs, and is expected to be a simple and effective prognostic evaluation indicator.

## Data Availability Statement

Publicly available datasets were analyzed in this study. This data can be found at: https://mimic.mit.edu.

## Ethics Statement

The studies involving human participants were reviewed and approved by the Institutional Review Boards of Massachusetts Institute of Technology (Cambridge, MA, USA) and Beth Israel Deaconess Medical Center (Boston, MA, USA). Written informed consent for participation was not required for this study in accordance with the national legislation and the institutional requirements.

## Author Contributions

ZY and LZ conceived and designed the study. YT and XZ collected and analyzed the clinical data. YT drafted the manuscript. YF and QC participated in the implementation of statistical methods in this study and put forward constructive suggestions. ZL and HL reviewed the study and participated in the interpretation of the results. All authors gave final approval of the version to be published and agree to be accountable for all aspects of the work.

## Funding

This study was supported by the National Natural Science Foundation of China (81873416 and 82100071), the Key Research and Development Program of Hunan Province (2020SK2065), and Natural Science Foundation Project of Hunan Province (2020JJ4634).

## Conflict of Interest

The authors declare that the research was conducted in the absence of any commercial or financial relationships that could be construed as a potential conflict of interest.

## Publisher's Note

All claims expressed in this article are solely those of the authors and do not necessarily represent those of their affiliated organizations, or those of the publisher, the editors and the reviewers. Any product that may be evaluated in this article, or claim that may be made by its manufacturer, is not guaranteed or endorsed by the publisher.

## Abbreviations

CHF: Congestive heart failure
SII: Systemic immune-inflammation index
MIMIC-III: Medical Information Mart for Intensive Care III
ICD-9: The ninth revision of the International Classification of Diseases
ICU: Intensive care unit
MACEs: Major adverse cardiac events
SOFA: The sequential organ failure assessment
SAPSII: The simplified acute physiology score II
SD: Standard deviation
GAM: Generalized additive model
K-M curve: Kaplan-Meier curve
BUN: Blood urea nitrogen
cTnT: Troponin T
NT-proBNP: N-terminal probrain natriuretic peptide
VIF: Variance inflation factor
PSM: Propensity score matching
VHD: Valvular heart disease
AMI: Acute myocardial infarction
TNF-α: Tumor necrosis factor α
IL-6: Interleukin 6
MPV: Mean platelet volume
FDR: False discovery rate.
