# Corrigendum: Association of systemic immune-inflammation index with short-term mortality of congestive heart failure: A retrospective cohort study

**DOI:** 10.3389/fcvm.2022.1116547

**Published:** 2022-12-19

**Authors:** Yiyang Tang, Xiaofang Zeng, Yilu Feng, Qin Chen, Zhenghui Liu, Hui Luo, Lihuang Zha, Zaixin Yu

**Affiliations:** ^1^Department of Cardiology, Xiangya Hospital, Central South University, Changsha, China; ^2^Department of Neurology, Xiangya Hospital, Central South University, Changsha, China; ^3^Department of Cardiovascular Medicine, the First Hospital of Changsha, Changsha, China; ^4^National Clinical Research Center for Geriatric Disorders (Xiang Ya), Changsha, China

**Keywords:** congestive heart failure, systemic immune-inflammation index, mortality, biomarker, MIMIC-III database

In the published article, there was an error in the Funding statement. The fund number (8210012237) was the initial acceptance number, not the final grant number. The correct Funding statement appears below.

