# Supplementary material for: Association of Systemic Immune-Inflammation Index With Short-Term Mortality of Congestive Heart Failure: A Retrospective Cohort Study
Source: Front Cardiovasc Med. 2021 Nov 12;8:753133. doi: 10.3389/fcvm.2021.753133 (PMC8632819; doi:10.3389/fcvm.2021.753133)
Supplement: Supplementary file 1 [file Table_1.DOCX]

**Supplementary Table 1.** The clinical characteristics of patients with CHF according to SII levels.

| **Parameter** | **SII** | | | ***P* value** |
| --- | --- | --- | --- | --- |
|  | **<1144.28** | **≥1144.28, <2730.11** | **≥2730.11** |  |

| **Demographics** |  |  |  |  |
| --- | --- | --- | --- | --- |
| Age, years | 73.3 (19.4-95.7) | 74.5 (22.9-90.3) | 76.4 (18.4-95.4) | <0.001 |
| Male, n (%) | 846 (55.7%) | 843 (53.8%) | 747 (49.1%) | 0.001 |
| Ethnicity, n (%) |  |  |  | <0.001 |
| White | 1080 (71.1%) | 1131 (72.2%) | 1138 (74.9%) |  |
| Black | 142 (9.3%) | 111 (7.1%) | 79 (5.2%) |  |
| Others | 298 (19.6%) | 324 (20.7%) | 303 (19.9%) |  |
| **Vital signs** |  |  |  |  |
| HR, beats/minute | 83.3 (45.2-135.7) | 84.5 (45.2-135.7) | 87.5 (45.2-135.7) | <0.001 |
| RR, times/minute | 18.8 (10.9-35.8) | 19.5 (10.9-35.8) | 20.0 (10.9-35.8) | <0.001 |
| SBP, mmHg | 114.0 (79.5-175.5) | 113.5 (79.5-175.5) | 113.4 (79.5-175.5) | 0.904 |
| DBP, mmHg | 57.4 (32.5-97.2) | 57.2 (32.5-97.2) | 56.4 (32.5-97.2) | 0.201 |
| Temperature, ℃  ◦ C | 36.8 (34.0-38.9) | 36.8 (34.0-38.9) | 36.7 (34.0-38.9) | 0.023 |
| SpO2, % | 97.4 (84.8-100.0) | 97.2 (84.8-100.0) | 97.1 (84.8-100.0) | <0.001 |
| Weight, kg | 78.4 (35.5-212.0) | 79.8 (35.5-212.0) | 75.9 (35.5-212.0) | <0.001 |
| **Therapies, n (%)** |  |  |  |  |

| ACEI | 408 (26.8%) | 430 (27.5%) | 428 (28.2%) | 0.719 |
| --- | --- | --- | --- | --- |
| ARB | 51 (3.4%) | 62 (4.0%) | 70 (4.6%) | 0.211 |
| β-blocker | 903 (59.4%) | 967 (61.7%) | 957 (63.0%) | 0.123 |
| Digoxin | 140 (9.2%) | 150 (9.6%) | 185 (12.2%) | 0.014 |
| Furosemide | 1025 (67.4%) | 1089 (69.5%) | 1076 (70.8%) | 0.128 |
| Statins | 479 (31.5%) | 505 (32.2%) | 467 (30.7%) | 0.660 |
| Dialysis | 143 (9.4%) | 164 (10.5%) | 171 (11.2%) | 0.247 |
| Vasopressor | 384 (25.3%) | 398 (25.4%) | 452 (29.7%) | 0.007 |
| Ventilation | 527 (34.7%) | 619 (39.5%) | 733 (48.2%) | <0.001 |
| Assisted circulation | 76 (5.0%) | 117 (7.5%) | 89 (5.9%) | 0.014 |

**Supplementary Table 1.** Continued.

| **Parameter** | **SII** | | | ***P* value** |
| --- | --- | --- | --- | --- |
|  | **<1144.28** | **≥1144.28, <2730.11** | **≥2730.11** |  |

| **Laboratory events** |  |  |  |  |
| --- | --- | --- | --- | --- |
| Hemoglobin, g/dl | 10.9 (5.3-17.3) | 11.1 (5.3-17.3) | 10.8 (5.3-17.3) | 0.002 |
| Creatinine, mg/dl | 1.2 (0.3-12.1) | 1.2 (0.3-12.1) | 1.2 (0.3-11.3) | 0.008 |
| BUN, mg/dl | 25.0 (4.0-153.0) | 27.0 (4.0-153.0) | 29.0 (4.0-153.0) | <0.001 |
| Glucose, mg/dl | 129.5 (66.0-374.9) | 135.7 (66.0-374.9) | 142.5 (66.0-374.9) | <0.001 |
| Sodium, mmol/L | 139.0 (118.0-159.0) | 139.0 (118.0-159.0) | 138.0 (118.0-159.0) | 0.008 |
| Potassium, mmol/L | 4.1 (2.5-8.0) | 4.2 (2.5-8.0) | 4.2 (2.5-8.0) | <0.001 |
| Chloride, mmol/L | 104.0 (81.0-129.0) | 103.0 (81.0-129.0) | 103.0 (81.0-129.0) | <0.001 |
| Bicarbonate, mmol/L | 25.0 (9.0-43.0) | 24.0 (9.0-42.0) | 24.0 (9.0-43.0) | <0.001 |
| PT, second | 14.3 (10.6-63.6) | 14.3 (10.6-63.6) | 14.4 (10.6-63.6) | 0.504 |
| APTT, second | 32.6 (11.2-150.0) | 31.1 (15.9-150.0) | 31.5 (18.5-150.0) | 0.001 |
| Lactate, mmol/L | 1.9 (0.5-14.0) | 1.8 (0.5-14.0) | 1.8 (0.6-14.0) | 0.708 |
| Albumin, g/dL  ◦ C | 3.0 (1.3-4.8) | 3.0 (1.3-4.8) | 2.9 (1.3-4.8) | <0.001 |
| Bilirubin, mg/dL | 1.6 (0.0-13.1) | 1.4 (0.0-13.1) | 1.1 (0.0-13.1) | <0.001 |
| ALT, IU/L | 67.0 (3.7-2040.0) | 60.4 (3.7-2040.0) | 52.0 (3.7-2040.0) | 0.002 |
| NT-proBNP, ng/ml | 10.92 (0.0-63.1) | 10.97 (0.0-63.1) | 11.00 (0.0-63.1) | 0.399 |
| cTnT, ng/mL | 1.4 (0.0-12.7) | 1.4 (0.0-12.7) | 1.2 (0.0-12.7) | 0.223 |
| CI, L/min/m^2^ | 2.8 (1.0-5.9) | 2.7 (1.0-5.9) | 2.8 (1.0-5.9) | 0.055 |
| Urine output, L | 1.7 (0.0-7.4) | 1.6 (0.0-7.4) | 1.4 (0.0-7.4) | <0.001 |
| **Hospitalization type** |  |  |  | 0.003 |
| Emergency, n (%) | 1312 (86.3%) | 1376 (87.9%) | 1374 (90.4%) |  |
| Elective, n (%) | 73 (4.8%) | 52 (3.3%) | 52 (3.4%) |  |
| **First ICU admission** |  |  |  | <0.001 |
| CCU, n (%) | 406 (26.7%) | 436 (27.8%) | 394 (25.9%) |  |
| MICU, n (%) | 619 (40.7%) | 643 (41.1%) | 743 (48.9%) |  |

**Supplementary Table 1.** Continued.

| **Parameter** | **SII** | | | ***P* value** |
| --- | --- | --- | --- | --- |
|  | **<1144.28** | **≥1144.28, <2730.11** | **≥2730.11** |  |

| **Comorbidities, n (%)** |  |  |  |  |
| --- | --- | --- | --- | --- |
| Hypertension | 295 (19.4%) | 349 (22.3%) | 293 (19.3%) | 0.063 |
| Hyperlipemia | 427 (28.1%) | 470 (30.0%) | 363 (23.9%) | <0.001 |
| Diabetes mellitus | 535 (35.2%) | 593 (37.9%) | 529 (34.8%) | 0.154 |
| Atrial fibrillation | 629 (41.4%) | 726 (46.4%) | 722 (47.5%) | 0.001 |
| AMI | 84 (5.5%) | 129 (8.2%) | 133 (8.8%) | 0.001 |
| VHD | 131 (8.6%) | 170 (10.9%) | 162 (10.7%) | 0.075 |
| Peripheral vascular | 179 (11.8%) | 201 (12.8%) | 197 (13.0%) | 0.555 |
| Pulmonary circulation | 87 (5.7%) | 98 (6.3%) | 107 (7.0%) | 0.326 |
| Pneumonia | 366 (24.1%) | 441 (28.2%) | 543 (35.7%) | <0.001 |
| COPD | 42 (2.8%) | 76 (4.9%) | 127 (8.4%) | <0.001 |
| Liver diseases | 112 (7.4%) | 74 (4.7%) | 46 (3.0%) | <0.001 |
| Renal failure  ◦ C | 358 (23.6%) | 402 (25.7%) | 360 (23.7%) | 0.305 |
| Stroke | 73 (4.8%) | 93 (5.9%) | 84 (5.5%) | 0.371 |

| Hypothyroidism | 180 (11.8%) | 189 (12.1%) | 186 (12.2%) | 0.945 |
| --- | --- | --- | --- | --- |
| Malignancy | 66 (4.3%) | 78 (5.0%) | 109 (7.2%) | 0.002 |
| Depression | 127 (8.4%) | 110 (7.0%) | 122 (8.0%) | 0.355 |
| **Scores** |  |  |  |  |
| SOFA | 5.0 (0.0-21.0) | 5.0 (0.0-20.0) | 5.0 (0.0-18.0) | 0.061 |
| SAPSII | 39.0 (6.0-94.0) | 39.0 (10.0-100.0) | 43.0 (10.0-101.0) | <0.001 |
| **Length of ICU stay, h** | 83.5 (24.0-1654.0) | 93.0 (24.0-1824.0) | 109.0 (24.0-2403.0) | <0.001 |

Abbreviations: HR, heart rate; RR, respiratory rate; SBP, systolic blood pressure; DBP, diastolic blood pressure; SpO2, percutaneous oxygen saturation; ACEI, angiotensin-converting enzyme inhibitors; ARB, angiotensin receptor blockers; BUN, blood urea nitrogen; PT, prothrombin time; APTT, activated partial thromboplastin time; ALT, alanine transaminase; cTnT, troponin T; NT-proBNP, N-terminal pro brain natriuretic peptide; CI, cardiac index; SII, Systemic immune-inflammation index; CHF, congestive heart failure; AMI, acute myocardial infarction; VHD, valvular heart disease; COPD, chronic obstructive pulmonary disease. CCU, cardiac care unit; MICU, medical intensive care unit.
